# Determinants of 15‐Year Progression‐Free Survival in Multiple Myeloma; Real‐World Data From a Single Institution

**DOI:** 10.1002/ajh.70012

**Published:** 2025-07-18

**Authors:** Meletios A. Dimopoulos, Ioannis Ntanasis‐Stathopoulos, Despina Fotiou, Foteini Theodorakakou, Eirini Solia, Vasiliki Spiliopoulou, Panagiotis Malandrakis, Erasmia Psimenou, Stavroula Giannouli, Nikolaos Kanellias, Vassilis Koutoulidis, Maria Roussou, Angeliki Andrikopoulou, Magdalini Migkou, Evangelos Eleutherakis‐Papaiakovou, Maria Gavriatopoulou, Evangelos Terpos, Efstathios Kastritis

**Affiliations:** ^1^ Department of Clinical Therapeutics, School of Medicine National and Kapodistrian University of Athens Athens Greece; ^2^ Department of Medicine Korea University Seoul South Korea; ^3^ Second Department of Internal Medicine, School of Medicine National and Kapodistrian University of Athens Athens Greece; ^4^ First Department of Radiology, School of Medicine, Aretaieion Hospital National and Kapodistrian University of Athens Athens Greece

**Keywords:** chemotherapy, functional cure, multiple myeloma, novel agents, overall survival, progression‐free survival

## Abstract

The therapeutic advances during the last two decades have rendered multiple myeloma a chronic disease and, thus, it is important to identify patient subgroups which may have extremely favorable outcomes and optimize their treatment. The current study aimed to evaluate the clinical and disease characteristics of patients with very long follow‐up (minimum 15 years), to identify those with very long survival (> 15 years) and those with very long disease remissions (> 15 years) after frontline treatment diagnosed at a single center from 1994 to 2009. Among 323 consecutive, unselected patients, the calculated 15‐year and 20‐year cumulative survival rates were 18% and 14%, respectively. Forty‐nine survived for more than 15 years. Furthermore, the calculated 15‐year and 20‐year cumulative PFS rates for the 323 patients were 9% and 7%, respectively; 25 (8%) patients were identified as long‐term progression‐free survivors. Younger age (≤ 65 years), good performance status (ECOG PS 0–1), low/intermediate risk stratification (ISS 1 or 2), absence of adverse cytogenetic abnormalities, autologous transplantation and achievement of CR to initial therapy were significantly associated with prolonged PFS. Importantly, all patients who were in complete response with negative minimal residual disease at 15 years remained at the same disease status at last follow‐up. In conclusion, among patients treated with either conventional chemotherapy or with first‐generation novel agents (thalidomide, bortezomib, or lenalidomide), about 15% were long‐term survivors and 8% remained in long‐term remission for more than 15 years without maintenance treatment.

## Graphical abstract

9% of patients with multiple myeloma achieve 15‐year progression‐free survival; Kaplan‐Meier curves for PFS (A) and OS (B).

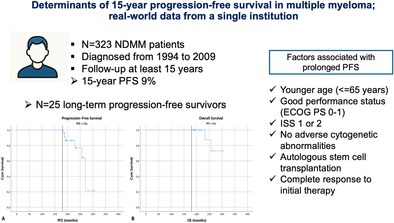

## 1 Introduction

Multiple myeloma (MM) is defined by the abnormal clonal expansion of plasma cells, resulting in organ damage, which presents as bone lesions, anemia, renal impairment, and hypercalcemia [1]. MM is a chronic disease for most patients, but it is associated with substantially shortened survival compared to age‐matched individuals [2]. Importantly, there has been a declining trend in MM‐specific mortality, especially in the last 25 years, probably attributable to the integration of innovative therapeutic agents in MM treatment [3, 4].

In recent decades, advancements in the treatment of MM have markedly extended both progression‐free survival (PFS) and overall survival (OS), especially in younger patients. A significant proportion of patients now achieve prolonged PFS, which challenges the conventional belief of the incurability of MM and introduces the idea of a “functional” or “operational” cure [5, 6, 7]. This concept denotes a significantly prolonged PFS that corresponds with longer OS and death rates approaching those of the general population. Prolonged PFS after upfront treatment is more closely associated with “functional” cure, since the length of PFS (and OS) decreases with each additional line of therapy [8, 9].

It is important, however, to identify subgroups which may have extremely favorable outcomes and which could help to further refine MM therapeutic approaches and potential alternative treatment strategies. Yet, the demonstration of long‐term benefit requires prolonged follow‐up. Historical cohorts are useful for the assessment of new therapies and regimens that are being integrated into clinical practice, allowing the evaluation of long‐term outcomes in an unselected patient population, as compared to clinical trials [10, 11].

The current study aimed to describe the clinical and disease characteristics of patients with very long follow up (minimum of at least 15 years), to identify patients with very long survival (i.e., > 15 years) and within them those with very long disease remissions after frontline treatment treated at a single center during the same time period.

## 2 Patients and Methods

The analysis included consecutive, unselected, newly diagnosed patients with MM (NDMM) who initiated first‐line therapy at the Department of Clinical Therapeutics (Athens, Greece) between January 1994 and December 2009. This ensured that all eligible patients had a minimum follow‐up of at least 15 years from the start of primary therapy. All patients had symptomatic disease based on the International Myeloma Working Group criteria of the time, requiring at least one CRAB symptom to initiate anti‐myeloma therapy. Patient and disease characteristics were recorded in a prospectively maintained database, and the Institutional Scientific/Ethics committee has granted approval for data analysis and publication.

The primary outcomes of interest were PFS and OS. PFS was defined as the interval between treatment initiation and either disease progression or death from any cause. OS was defined as the interval between the date of treatment initiation and death from any cause. Patients without documented progression or death were censored at the time of their last known follow‐up.

For statistical analysis, the chi‐square test, along with Fisher's exact test when required, was used to compare categorical variables between groups. The Mann–Whitney test was applied for comparisons of quantitative variables. Multivariate Cox regression analysis was performed to identify prognostic factors associated with time‐to‐event outcomes. A logistic regression analysis including variables that emerged as statistically significant in the univariate analysis was also performed to determine predictors of long survival. A two‐sided *p* of less than 0.05 was considered statistically significant. All statistical analyses were conducted using IBM SPSS v.24 (SPSS Inc., Chicago, IL).

## 3 Results

### 3.1 Long‐Term Survivors (More Than 15 Years)

This study examined the characteristics of patients with multiple myeloma who survived over 15 years since they started treatment, comparing them to the broader cohort treated at the same institution. For the entire cohort of 323 patients, the calculated 15‐year and 20‐year cumulative survival rates (adjusting for censoring) were 18% and 14%, respectively. Among all patients, 49 achieved a survival time of more than 15 years.

Table 1 shows the characteristics of the long‐term survivors for more than 15 years since MM first line treatment (*n* = 49) compared to those with OS of 15 years or less (*n* = 274) and the whole patient cohort (*n* = 323). The long‐term survivors were significantly younger than the others at the time of initial therapy, with a median age of 52 years compared to 71 years. Notably, only 10.6% of long‐term survivors were older than 65 years, a sharp contrast to 68.8% among the others. Gender distribution was similar between groups, with females constituting 45% and 47% of the two subgroups, respectively.

**TABLE 1:** Characteristics of the long‐term survivors (*n* = 49, more than 15 years since MM first treatment) compared to those with OS 15 years or less (*n* = 274) and the whole patient cohort (*n* = 323).

Variable	All patients (*n* = 323)	Long‐term survivors OS > 15 y (*n* = 49, 15.2%)	Patients with OS ≤ 15 y (*n* = 274)	*p* (OS > 15 y vs ≤ 15 y)
Age (y)	69 (26–92)	52 (26–75)	71 (30–92)	< 0.001
Age > 65 y	181 (59.7%)	5 (10.6%)	176 (68.8%)	
Females	152 (47%)	22 (45%)	130 (47.4%)	0.74
PS at diagnosis	1 (0–4)	1 (0–4)	2 (0–4)	
PS > 1	152 (47%)	12 (25%)	140 (52%)	0.002
High‐risk cytogenetics at diagnosis^a^	27 (29%)	2 (14.3%)	25 (31.6%)	0.187
b2 microglobulin (mg/L)	4.1 (1.1–52.2)	2.5 (1.1–15)	4.6 (1.1–52.2)	
< 3.5 mg/L	130 (40.9%)	37 (77.1%)	93 (34.4%)	
3.5–5.5 mg/L	73 (23%)	6 (12.5%)	67 (24.8%)	
> 5.5 mg/L	115 (36.2%)	5 (10.4%)	110 (40.7%)	< 0.001
Serum albumin (g/dL)	3.6 (1.3–5.5)	4 (1.7–5.5)	3.5 (1.3–5.1)	
Serum albumin < 3.5 g/dL	148 (46.3%)	15 (32%)	133 (49%)	0.033
Serum LDH (IU/L)	180 (72–596)	180 (88–370)	180 (72–596)	
LDH abnormal high	110 (34.6%)	13 (27.7%)	97 (35.8%)	0.28
Serum calcium—mg/dL	9.6 (7–18.7)	9.6 (7.6–12.8)	9.6 (7–18.7)	
Hypercalcemia	56 (17.4%)	3 (6.3%)	53 (19.3%)	0.027
Hemoglobulin – g/dL	10.1 (4.1–15.3)	11.7 (8–15.3)	10 (4.1–15.3)	
Anemia (Hb < 10 g/dL)	135 (41.8%)	7 (14.3%)	128 (46.7%)	< 0.001
PLTs (×10^9^/L)	210 (35–686)	250.5 (109–591)	200 (35–686)	
PLTs < 130 × 10^9^/L	39 (12.1%)	2 (4.1%)	37 (13.5%)	0.066
Serum creatinine (mg/dL)	1.1 (0.4–12.8)	0.9 (0.5–12.5)	1.1 (0.4–12.8)	
eGFR (mL/min)	64.4 (3.9–163.9)	72.2 (4.5–133)	60.2 (3.9–163.9)	
Severe RI (eGFR < 30 mL/min)	61 (20%)	4 (8.5%)	57 (22.4%)	0.030
Involved heavy chain				
IgG	175 (54.2%)	31 (63%)	144 (52.6%)	
IgA	87 (26.9%)	8 (16%)	79 (28.8%)	
IgD	2 (0.6%)	1 (2%)	1 (0.4%)	
None	57 (17.6%)	9 (18%)	48 (17.5%)	0.35
Involved light chain				
Kappa	166 (51%)	25 (51%)	141 (52%)	
Lambda	114 (35%)	18 (37%)	96 (35%)	0.97
ISS at baseline				
1	83 (26.2%)	25 (53.2%)	58 (21.5%)	
2	119 (37.5%)	17 (36.2%)	102 (37.8%)	< 0.001
3	115 (36.3%)	5 (10.6%)	110 (40.7%)	
R‐ISS at baseline				
1	11 (8%)	1 (6%)	10 (8.3%)	
2	62 (45%)	12 (70%)	50 (41.7%)	0.078
3	64 (47%)	4 (24%)	60 (50%)	
Induction therapy type				0.19
Steroids	10 (3.1%)	1 (2%)	9 (3.3%)	
Chemo‐Dexa	131 (40.6%)	25 (51%)	106 (38.7%)	
Thal‐based ± Chemo	91 (28.2%)	11 (22.4%)	80 (29.2%)	
VTd/VRd	18 (5.6%)	3 (6.1%)	15 (5.5%)	
Bortezomib‐based ± Chemo	24 (7.4%)	6 (12.2%)	18 (6.6%)	
Rd/MPR	49 (15.2%)	3 (6.1%)	46 (16.8%)	
HDM/ASCT	87 (26.9%)	35 (71.4%)	52 (19%)	< 0.001
Consolidation	19 (5.9%)	8 (16.3%)	11 (4%)	< 0.001
Maintenance	41 (12.7%)	18 (36.7%)	23 (8.4%)	< 0.001
Median OS (months, 95% CI)	51.8 (44.1–59.5)	NC (only 6 events)	44.2 (38.9–49.5)	
Median PFS (months, 95% CI)	21.1 (18.5–23.7)	185.3 (84.4–285.1)	19 (16.7–21.3)	

*Note*: All continuous variables are presented as median (range) and all categorical variables as *n* (%).

Abbreviations: Chemo: conventional chemotherapy; CI: confidence interval; Dexa: dexamethasone; eGFR: estimated glomerular filtration rate according to Cockroft‐Gault; HDM/ASCT: high dose melphalan/autologous stem cell transplantation; MPR: melphalan, prednisone, lenalidomide; NC: not calculable; OS: overall survival; PFS: progression‐free survival; PLTs: platelets; PS: ECOG performance status; Rd: lenalidomide, dexamethasone; (R)ISS: (revised) international staging system; Thal: Thalidomide; VRd: bortezomib, lenalidomide, dexamethasone; VTd: bortezomib, thalidomide, dexamethasone; y: years.

^a^ Data on del17p, t(4;14) and t (14;16) available only in 93 patients.

Several baseline disease characteristics highlighted more favorable prognostic factors among long‐term survivors. Only one quarter of the patients in the long OS group had an Eastern Cooperative Oncology Group Performance Status (ECOG PS) 2–4, compared to half of the patients in the other group (*p* = 0.002). Severe renal impairment was present in only 8.5% of long‐term survivors compared to 22.4% in the other group (*p* = 0.03); hypercalcemia and anemia were also significantly less frequent with rates of 6.3% and 14.3%, versus 19.3% (*p* = 0.03) and 46.7% (*p* < 0.001) in the other group, respectively.

Serum beta‐2 microglobulin levels, an important prognostic marker, were markedly lower in long‐term survivors, with 77.1% having levels below 3.5 mg/L compared to 34.4% among the others (*p* < 0.001). Furthermore, serum albumin levels were higher in long‐term survivors, with only 32% presenting with levels below 3.5 g/dL compared to 49% (*p* = 0.03). Thus, patient stratification according to the International Staging System (ISS) differed significantly between long‐term survivors and those with OS less than 15 years (*p* < 0.001). More long‐term survivors were diagnosed with ISS stage 1 disease (53.2% vs. 21.5%, respectively) and fewer at stage 3 (10.6% vs. 40.7%, respectively). Among those with available data for cytogenetics, high‐risk cytogenetics were less frequent among long‐term survivors (14.3% versus 31.6%, respectively), although this difference did not reach statistical significance. Similarly, the Revised ISS (R‐ISS) staging did not significantly differ between these groups, probably due to the small number of patients with available data.

All patients received treatment with steroids and either conventional chemotherapy or immunomodulatory drugs (thalidomide, lenalidomide) or first‐generation proteasome inhibitors (bortezomib) and few of their combinations (Table 1). Treatment patterns revealed a strong association between long‐term survival and the use of high‐dose melphalan with autologous stem cell transplantation as part of 1st line therapy, which was employed in 71.4% of long‐term survivors compared to 19% among the others (*p* < 0.001). Additionally, long‐term survivors received maintenance therapies more frequently (*p* < 0.001). There was no statistically significant difference in the median PFS or OS between those diagnosed before and after 2003. Importantly, immunological recovery, in terms of normalization of uninvolved immunoglobulin levels, was observed in 43/49 long survivors (88%). Regarding first salvage treatments for patients who experienced long OS but had a disease relapse, 11 patients received PI‐based regimens (Vd, Kd, and VCd), 9 patients received IMiD‐based doublets (Rd and Thal‐dex), 5 patients received PI‐ and IMiD‐based regimens (VRd, Ixazomib‐Rd, VMP‐T) and 2 patients received chemotherapy‐based treatment at first relapse. In the subsequent lines of therapy, all but one patient received lenalidomide‐based combinations, eight patients received anti‐CD38‐based combinations, two patients received elotuzumab‐based regimens, two patients received anti‐BCMA bispecific antibody, and one patient received belantamab mafodotin.

**FIGURE 1:**
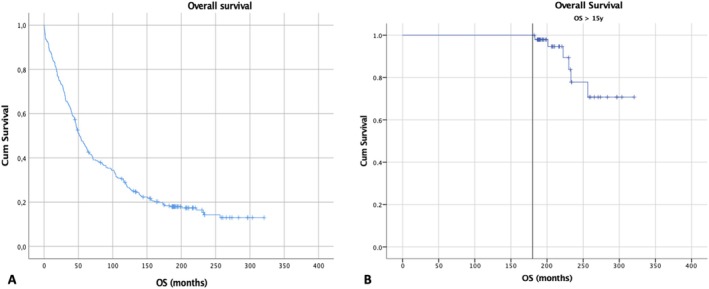
OS Kaplan–Meier curves for the entire cohort (*n* = 323) (A) and the long‐term OS group > 15 years (*n* = 49) (B). The vertical reference line denotes the 15‐year (180 months) landmark. [Color figure can be viewed at wileyonlinelibrary.com]

**FIGURE 2:**
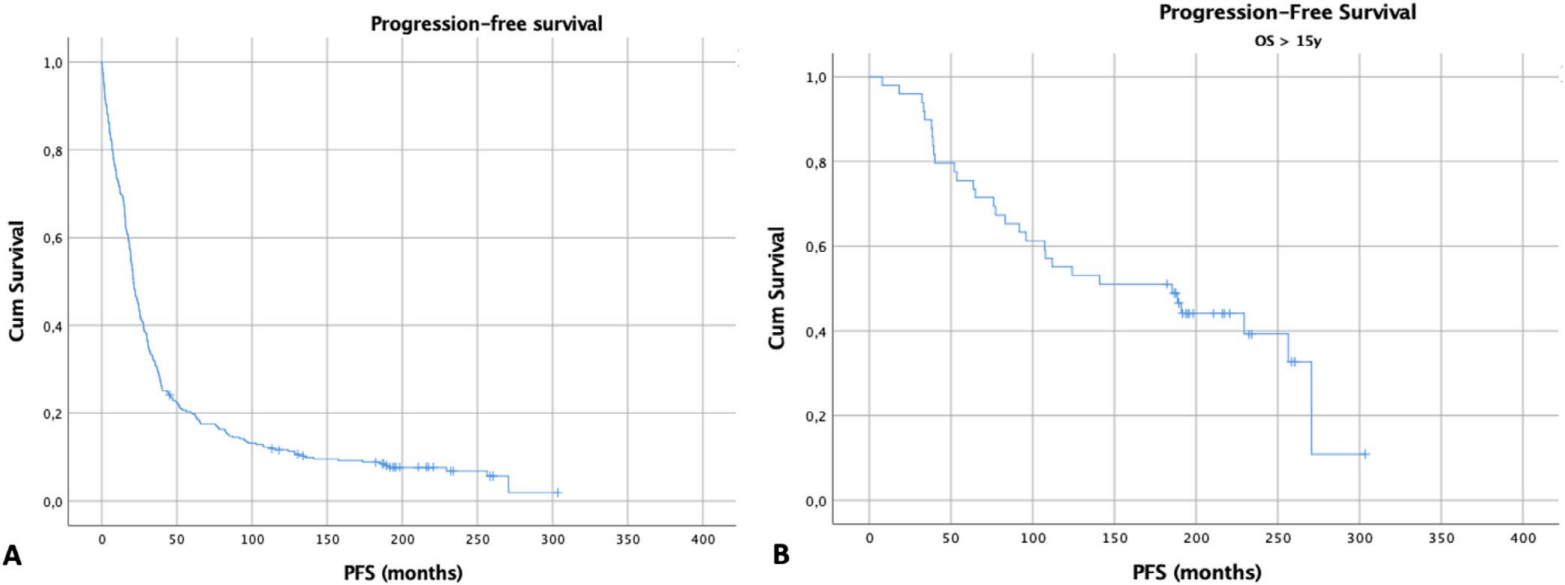
PFS Kaplan–Meier curves for the entire cohort (*n* = 323) [A] and the long‐term OS group > 15 years (*n* = 49) (B). [Color figure can be viewed at wileyonlinelibrary.com]

Median OS was not calculable in long‐term survivors due to the limited number of events, while median OS in the broader cohort was 4.3 years (Figures 1 and 2). In the multivariate Cox regression analysis of baseline characteristics for OS, younger age (≤ 65 years) (*p* < 0.001), ISS stage 1 (*p* < 0.001), good PS (0–1) (*p* = 0.001) and normal calcium levels (*p* = 0.003) at diagnosis were significantly correlated with favorable OS. In the logistic regression analysis of factors predicting for long OS > 15 years, younger age (≤ 65 years) (*p* < 0.001), low levels of serum b2‐microglobulin (< 3.5 mg/L) (*p* = 0.042), hemoglobin ≥ 10 g/dL (*p* = 0.016) and use of high‐dose melphalan followed by autologous stem cell transplantation (*p* = 0.035) were significantly associated with long OS.

### 3.2 Long‐Term Remissions (More Than 15 Years)

At the last follow‐up, 36.7% (*n* = 18) of long‐term survivors remained progression‐free. Median PFS in the long‐term survivor group was 15.4 years compared to 1.8 years among those with OS less than 15 years.

The calculated 15‐year and 20‐year cumulative PFS rate (adjusting for censoring) was 8% and 7%, respectively, for the entire cohort of 323 patients. Among them, 25 were identified as long‐term progression‐free survivors (PFS more than 15 years).

Table 2 shows the characteristics of the long‐term progression‐free survivors for more than 15 years since MM diagnosis (*n* = 25) compared to those with PFS of 15 years or less (*n* = 298). The median age of the long‐term PFS group was significantly lower than the others (53 vs. 70 years; *p* < 0.001). Additionally, only 16.7% of the long‐term PFS group were aged over 65 years, compared to 63.4% among the others. Gender distribution was similar across groups, with females comprising 48% of the long‐term PFS group and 47% of the others, respectively.

**TABLE 2:** Characteristics of the long‐term non‐progressors (*n* = 25, more than 15 years since MM first treatment) compared to those with PFS 15 years or less (*n* = 298).

Variable	Long‐term PFS > 15 y (*n* = 25, 7.7%)	Patients with PFS ≤ 15 y (*n* = 298)	*p*
Age (y)	53 (26–75)	70 (30–92)	< 0.001
Age > 65 y	4 (16.7%)	177 (63.4%)	
Females	12 (48%)	140 (47%)	0.92
PS at diagnosis	1 (0–3)	2 (0–4)	
PS > 1	5 (20%)	147 (49%)	0.005
High‐risk cytogenetics at diagnosis^a^	0	27 (30.3%)	0.19
b2 microglobulin (mg/L)	2.4 (1.1–15)	4.4 (1.1–52.2)	
< 3.5 mg/L	21 (87.5%)	109 (37.1%)	
3.5–5.5 mg/L	1 (4.2%)	72 (24.5%)	
> 5.5 mg/L	2 (8.3%)	113 (38.4%)	< 0.001
Serum albumin (g/dL)	4 (1.7–5.5)	3.5 (1.3–5.2)	
Serum albumin < 3.5 g/dL	8 (33.3%)	140 (47.3%)	0.19
Serum LDH (IU/L)	183.5 (97–370)	180 (72–596)	
LDH abnormal high	6 (25%)	104 (35.4%)	0.30
Serum calcium (mg/dL)	9.4 (7.6–12.8)	9.7 (7–18.7)	
Hypercalcemia	2 (8%)	54 (18.2%)	0.20
Hemoglobulin (g/dL)	11.5 (8–15.3)	10.1 (4.1–15.3)	
Anemia (Hb < 10 g/dL)	2 (8%)	133 (44.6%)	< 0.001
PLTs (×10^9^/L)	262 (129–591)	205 (35–686)	
PLTs < 130 × 10^9^/L	1 (4.2%)	38 (12.8%)	0.19
Serum creatinine (mg/dL)	0.9 (0.5–7.7)	1.1 (0.4–12.8)	
eGFR (mL/min)	72.9 (7.5–133.2)	61.6 (3.9–163.9)	
Severe RI (eGFR < 30 mL/min)	1 (4.2%)	60 (21.6%)	0.04
Involved Heavy chain [n (%)]			
IgG	15 (60%)	160 (53.7%)	
IgA	4 (16%)	83 (27.9%)	
IgD	1 (4%)	1 (0.3%)	
None	5 (20%)	52 (17.4%)	0.25
Involved light chain			
Kappa	12 (48%)	154 (51.7%)	
Lambda	10 (40%)	104 (34.9%)	0.95
ISS at baseline			
1	15 (62.5%)	68 (23.2%)	
2	7 (29.2%)	112 (38.2%)	< 0.001
3	2 (8.3%)	113 (38.6%)	
R‐ISS at baseline			
1	1 (16.7%)	10 (7.6%)	
2	3 (50%)	59 (45%)	0.65
3	2 (33.3%)	62 (47.3%)	
Induction therapy type			
Steroids	0	10 (3.4%)	
Chemo‐Dexa	14 (56%)	117 (39.3%)	
Thal‐based ± Chemo	5 (20%)	86 (28.9%)	
VTd/VRd	2 (8%)	16 (5.4%)	0.51
Bortezomib‐based ± Chemo	2 (8%)	22 (7.4%)	
Rd/MPR	2 (8%)	47 (15.8%)	
HDM/ASCT	15 (60%)	72 (24.2%)	< 0.001
Consolidation	4 (16%)	15 (5%)	0.065
Maintenance	9 (36%)	32 (10.7%)	< 0.001
Median OS (months, 95% CI)	NC (only 2 events)	46.9 (41.3–52.6)	
Median PFS (months, 95% CI)	270.6 (244.4–296.8)	19.9 (18.2–21.6)	

*Note*: All continuous variables are presented as median (range) and all categorical variables as *n* (%).

Abbreviations: Chemo: conventional chemotherapy; CI: confidence interval; Dexa: dexamethasone; eGFR: estimated glomerular filtration rate according to Cockroft‐Gault; HDM/ASCT: high dose melphalan/autologous stem cell transplantation; MPR: melphalan, prednisone, lenalidomide; NC: not calculable; OS: overall survival; PFS: progression‐free survival; PLTs: platelets; PS: ECOG performance status; Rd: lenalidomide, dexamethasone; (R)ISS: (revised) international staging system; Thal: Thalidomide; VRd: bortezomib, lenalidomide, dexamethasone; VTd: bortezomib, thalidomide, dexamethasone; y: years.

^a^ Data on del17p, t(4;14) and t (14;16) available only in 93 patients.

Only one fifth of the patients in the long‐term PFS group had ECOG PS 2–4, compared to approximately half of the patients in the other group (*p* = 0.005). A significant difference was also observed in the distribution of β2‐microglobulin levels: 87.5% of long‐term PFS patients had levels < 3.5 mg/L, compared to 37.1%. Rates of anemia (8% vs. 44.6%, respectively, *p* < 0.001) and severe renal impairment (4.2% vs. 21.6%, respectively, *p* = 0.04) were significantly lower in the long‐term PFS group. Hypercalcemia and high LDH levels were less frequent in the long‐term PFS group, but these differences were not statistically significant.

The stratification per ISS at diagnosis revealed a higher prevalence of ISS 1 disease among long‐term PFS patients (62.5% vs. 23.2%, respectively, *p* < 0.001). Baseline cytogenetics showed a lower proportion of high‐risk abnormalities in the long‐term PFS group (0% vs. 30%, respectively), although this difference did not reach statistical significance due to low numbers of available data within each group. It must be noted that no patient in the long‐term PFS group had extramedullary plasmacytoma at diagnosis.

Regarding the type of treatment, a significantly greater proportion of long‐term PFS patients received upfront high‐dose melphalan followed by autologous stem cell transplantation (60% vs. 24.2%, respectively, *p* < 0.001). Maintenance therapy was also more commonly administered in the long PFS group compared to the others (36% vs. 10.7%, respectively, *p* < 0.001). The median (range) duration of maintenance was 5.2 (0.4–14.8) years. All patients but one interrupted maintenance treatment at some time during the disease course. The median time without treatment for the patients with long‐term PFS was 12 years (range: 0–22 years; interquartile range: 8.5–17 years) (Table 3).

**TABLE 3:** Detailed patient characteristics of the 25 non‐progressors at 15 years since MM diagnosis.

ID	Gender	Age at diagnosis (years)	ISS	First line treatment	Time off treatment (years)	PFS (years)	OS (years)	Status at 15 years	Status at last FUP
15PFS1	M	50	1	MP/Mdex	19	21.4	21.4	CR	Dead, unrelated to MM
15PFS2	M	44	1	CycloDex, INF‐a, HDM/ASCT, Methylprednisolone maintenance	5	25.3	25.3	VGPR, MRD positive	VGPR, MRD positive
15PFS3	F	58	1	VBMCP + INF‐a, HDM/ASCT, INF‐a maintenance	6	22.5	24.7	CR	CR, MRD negative on 2nd line treatment (VRd)
15PFS4	M	70	1	MP/Mdex	18	19.1	19.4	CR	Dead, due to MM 1st relapse
15PFS5	F	36	1	VAD	21	21.5	21.5	CR	CR
15PFS6	M	26	1	VAD, Thal maintenance	17	21.7	21.7	CR, MRD negative	CR, MRD negative
15PFS7	M	52	1	VAD, HDM/ASCT, Thal‐Dex maintenance	5	16.2	16.2	CR	CR
15PFS8	F	52	1	VAD	22	22.6	22.6	PR	PD, > 10% increase in the bone marrow plasma cells
15PFS9	F	52	1	VAD + Thal, HDM/ASCT	19	19.5	19.5	VGPR	VGPR
15PFS10	F	64	2	VAD, HDM/ASCT	17	18.1	18.1	CR, MRD positive	CR, MRD positive
15PFS11	M	49	3	VAD, HDM/ASCT	16	16.3	16.3	CR	CR
15PFS12	M	59	2	Thal‐Dex, HDM/ASCT, Thal‐Dex consolidation	0	15.4	17.3	PR	VGPR on 2nd line treatment (Rd)
15PFS13	M	43	2	PAD, HDM/ASCT, VTD consolidation	13	18.0	18.0	CR, MRD negative	CR, MRD negative
15PFS14	M	37	1	VAD, HDM/ASCT, VTD consolidation	10	18.4	18.4	CR, MRD negative	CR, MRD negative
15PFS15	M	46	1	CTD, HDM/ASCT	11	17.6	17.6	VGPR	VGPR
15PFS16	F	48	2	VTD, HDM/ASCT, Thal‐Dex maintenance	14	15.7	15.9	VGPR, MRD positive	PR on 2nd line treatment (Rd)
15PFS17	F	56	1	VTD, HDM/ASCT, VTD maintenance	13	16.5	16.5	CR	CR, PET/CT negative
15PFS18	M	54	2	CTD, HDM/ASCT, VTD maintenance	12	15.6	15.6	CR, MRD negative	CR, MRD negative
15PFS19	F	49	1	Vd, HDM/ASCT, VTD consol	11	15.6	15.6	CR, MRD negative	CR, MRD negative
15PFS20	F	75	1	MP/Mdex	12	15.8	15.8	PR	PR
15PFS21	M	66	2	Rd	7	15.2	15.2	PR	VGPR
15PFS22	F	70	1	MP/Mdex	11	16.0	16.0	PR	PR
15PFS23	F	65	2	MPR, Lenalidomide maintenance	6	16.1	16.1	CR, MRD negative	CR, MRD negative
15PFS24	M	54	3	VAD, AlloSCT	15	15.9	17.4	CR	PR on 2nd line treatment (VRd)
15PFS25	F	65	1	VAD, HDM/ASCT, Thal/INF‐a maintenance	11	19.4	19.4	CR, MRD negative	CR, MRD negative

Abbreviations: AlloSCT: allogeneic stem cell transplantation with a fully matched sibling; CR: complete response; CTD: cyclophosphamide, thalidomide, dexamethasone; CycloDex: cyclophosphamide, dexamethasone; Dex: dexamethasone; F: female; FUP: follow‐up; HDM/ASCT: high dose melphalan/autologous stem cell transplantation; INF‐a: interferon alfa‐2b; ISS: international staging system; M: male; Mdex: melphalan, dexamethasone; MM: multiple myeloma; MP: melphalan, prednisone; MPR: melphalan, prednisone, lenalidomide; MRD: minimal residual disease; OS: overall survival; PAD: bortezomib, doxorubicin, dexamethasone; PD: progressive disease; PET/CT: positron emission tomography–computed tomography; PFS: progression‐free survival; PR: partial response; Rd: lenalidomide, dexamethasone; Thal: thalidomide; VAD: vincristine, pegylated liposomal doxorubicin, dexamethasone; VBMCP: vincristine, carmustine, melphalan, cyclophosphamide, prednisone; Vd: bortezomib, dexamethasone; VGPR: very good partial response; (V)Rd: (bortezomib) lenalidomide, dexamethasone; VTD: bortezomib, thalidomide, dexamethasone.

At the 15‐year landmark, 16 out of the 25 patients (64%) were in complete remission (CR), and 8 were evaluated for MRD, with 7 of those having undetectable minimal residual disease (MRD) and 1 with detectable MRD. Among the remaining patients, 4 (16%) were in very good partial response (VGPR) and 5 (20%) in partial response (PR). Furthermore, immunological recovery, in terms of normalization of immunoglobulin level, was observed in 23/25 patients (92%).

One patient died due to reasons unrelated to MM; another patient died due to MM at first relapse. The other five patients had progressed and were alive; four were receiving second‐line treatment with bortezomib, lenalidomide, dexamethasone (VRd) or lenalidomide, dexamethasone (Rd) and were in clinical and hematologic response. The median (range) duration of response to second‐line treatment was 25 (13–36) months. All 7 patients with MRD negative CR have remained at the same disease status without treatment (Table 3).

**FIGURE 3:**
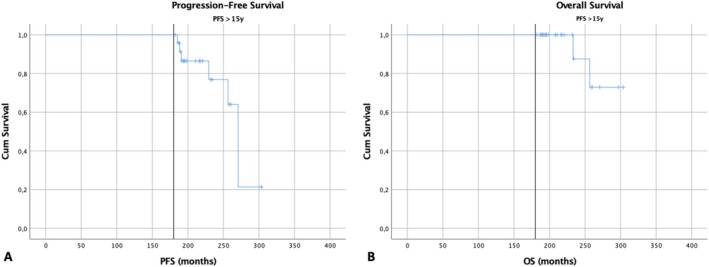
PFS (A) and OS (B) Kaplan–Meier curves for the long‐term PFS group > 15 years (*n* = 25). The vertical reference line denotes the 15‐year (180 months) landmark. [Color figure can be viewed at wileyonlinelibrary.com]

The median PFS for the long‐term PFS group was 22.6 years compared to 1.7 years for those with PFS ≤ 15 years. The median OS was not calculable in the long‐term PFS group as only 2 events were recorded (Figure 3). In the multivariate Cox regression analysis of baseline characteristics for long‐term PFS, younger age (≤ 65 years) (*p* < 0.001), ISS stage 1 (*p* = 0.007), hemoglobin ≥ 10 g/dL (*p* = 0.017) and normal calcium levels (*p* = 0.013) at diagnosis were significantly correlated with prolonged PFS.

## 4 Discussion

Our analysis, based on prospectively collected data on consecutive NDMM patients, showed that approximately 8% of patients achieved a prolonged PFS of at least 15 years in the era of conventional chemotherapy and first‐generation novel anti‐myeloma agents. To the best of our knowledge, this is the first study in the field that evaluated patients with a minimum follow‐up of 15 years. Furthermore, we assessed both OS and PFS among all patients who initiated treatment in our Unit and, thus, our data reflects real‐world conditions. Most prospective trials do not report long‐term outcomes. Furthermore, patients included in clinical trials are not representative of real‐world myeloma patients; very old patients, those with severe renal impairment, with severely impaired PS, or with comorbidities are typically excluded from clinical trials [12, 13, 14, 15, 16]. In our prospectively maintained database, we include all patients with symptomatic MM who receive frontline therapy, even those who receive only a single dose of treatment.

The main characteristics of our patients in the long PFS group of at least 15 years include young age (less than 65 years), good performance status (ECOG PS 0–1), low risk/low tumor burden disease (ISS 1 or 2), absence of adverse cytogenetic abnormalities, and achievement of CR to initial therapy. Furthermore, the majority of those patients showed an immunological recovery with sustained normalization of uninvolved immunoglobulin levels, which is important for attenuating the infection risk in the long term. Our results are consistent with a recent independent study evaluating the characteristics of patients achieving a PFS of at least 8 years following autologous stem cell transplantation [17]. These factors have also been recognized as favorable predictors for 10‐year OS in older studies [18, 19, 20, 21]. Low ISS stage and absence of high‐risk chromosomal aberrations remain well‐established features for favorable patient outcomes and prolonged survival during recent years irrespective of the advancements in MM therapeutics [22, 23, 24, 25, 26, 27]. Most, but not all, patients had also received high‐dose therapy and autologous stem cell transplantation followed by consolidation and/or fixed duration maintenance as part of the initial treatment. Several randomized clinical studies have shown the advantage of autologous stem cell transplantation over no transplant for PFS, both throughout the conventional chemotherapy period and after the introduction of first‐generation new anti‐myeloma drugs [28, 29, 30, 31, 32, 33]. Similarly, previous real‐world reports have also underscored the value of consolidating the response to first‐line anti‐myeloma therapy with high‐dose melphalan and autologous stem cell transplantation followed by consolidation and/or maintenance [34, 35, 36, 37, 38]. The optimization of induction, consolidation, and maintenance treatments has improved long‐term outcomes, especially after 2014 compared to the 90s, whereas it has been estimated that up to a fifth of the patients undergoing autologous transplant may now attain their normal life expectancy despite MM diagnosis [39]. In this cohort with a minimum follow‐up of 15 years and including patients between 1994 and 2009, only a minority of patients received induction with novel (at that time) drug triplet combinations. Most of our long‐term PFS patients received chemotherapy‐based regimens as induction treatment, as the novel anti‐myeloma therapies had not been approved for this use at that time, while thalidomide‐based regimens were available for older patients who were not eligible for and did not undergo ASCT. Tandem autologous and autologous‐allogeneic transplantation has been shown to deepen myeloma response and improve survival outcomes by achieving a median OS of at least 6 and 8 years, respectively [40, 41]. In our cohort of patients with long‐term PFS, only 1 patient underwent allogeneic stem cell transplantation; therefore, the exact clinical impact cannot be determined precisely. However, patient selection is crucial due to substantial treatment‐related toxicity.

It is important to consider that autologous transplant cannot be deemed a definitive assurance of prolonged PFS, given that up to 15% of patients may have an early recurrence within 12 months after transplant, whereas this percentage may reach 38% within 24 months [42, 43]. Furthermore, we cannot exclude the possibility that the effect of transplantation may serve as a proxy for age or patient fitness in our analysis, since the patients with long PFS were younger and had less common severe anemia and renal impairment. The latter in particular has been identified as an adverse prognostic factor in patients with MM [44]. This may also be the case in other studies in the literature reporting long‐term survival outcomes in younger patients with MM who received upfront autologous transplant [35, 36].

The long‐term sustainability of remissions even after the cessation of any anti‐myeloma treatment paves the way to curing myeloma, although there are fundamental differences between “operational” or “functional” cure and true curability [6, 45]. Achieving a cure in MM requires a cohort of patients who stay free of the illness, with a yearly mortality rate from all causes comparable to that of a normal population with the same sex and age distribution. In comparison to other curable hematologic malignancies [2], late relapses do occur in MM even after years in remission [37]. This was also confirmed in our data, as 6 out of the 25 patients with long‐term PFS relapsed after 15 years in remission.

Although sustainability of response, regardless of its depth, has been associated with prolonged OS [37, 46], it seems that the depth of response may differentiate the risk of relapse. Actually, we may define two groups of patients with long‐term PFS. In the first group, there are patients with detectable residual myeloma cells that may develop an immune equilibrium resembling smoldering myeloma or monoclonal gammopathy of undetermined significance. Nonetheless, this does not ensure an OS comparable to the general population [47, 48]. Interestingly, a recent study showed that the immune microenvironment of patients with MM, who remain in long‐term remission, presents persistent alterations following upfront treatment [49]. In patients with persistent or re‐emergent residual myeloma cells, these immunological changes were often associated with an inflammatory immune pathway that may ultimately disrupt the immune surveillance and lead to disease relapse. However, patients with no detectable myeloma cells had also persistent immunological changes probably related to prior exposure to myeloma and anti‐myeloma treatments.

In the other group of patients with long‐term PFS, patients do not have detectable residual myeloma. Based on the currently available diagnostic modalities, this condition would be ideally characterized by sustained, “deep stringent” or “total” MRD negative CR and defined as bone marrow MRD negative at 10^−6^ by next generation flow cytometry and/or next generation sequencing, imaging MRD negative by functional imaging positron emission tomography–computed tomography (PET/CT) and/or diffuse‐weighted whole body magnetic resonance imaging (DW WBMRI), no circulating tumor cells by flow cytometry and no monoclonal protein by mass spectrometry. Our data advocate for such an approach, since all CR MRD negative patients at 15 years remained at the same disease status at last follow‐up. Of course, the time frame to declare cure under these assumptions will be a matter of debate and more data are necessary. Furthermore, a cured patient should be free from long‐term disease‐ or treatment‐related complications. Therefore, we should have a conceptual transition from continuous to fixed‐duration treatment for those patients. Interestingly, the long‐term results of the fixed duration Total Therapy IV without the use of anti‐CD38 monoclonal antibodies showed a 40% 10‐year PFS rate among patients with low risk MM based on the gene expression profile [50]. Although maintenance after autologous transplantation improves patient outcomes, longer duration of maintenance has not been associated with superior PFS [51]. In this context, MRD negativity has been associated with prolonged PFS and it seems to emerge as a valuable indicator for interrupting maintenance treatment in MM [52, 53, 54, 55].

Among the limitations of our study, we should note the heterogeneity in clinical practice throughout the years. The study period spans a 30‐year time frame from 1994 to 2024. Both the diagnostic and therapeutic approaches of a newly diagnosed patient with MM have remarkedly changed. First‐generation novel agents bortezomib and lenalidomide gained regulatory approval for patients with relapsed/refractory multiple myeloma by the FDA in 2003 and 2006, respectively. Their integration into clinical practice, and especially in the frontline setting, was gradual over the years. For this reason, only 91/323 patients in our cohort had received first‐line treatment based on bortezomib and/or lenalidomide, and even fewer received their combinations. Therefore, our analysis is underpowered to reveal significant differences in survival outcomes between different time periods within the era under evaluation. Not all patients had available cytogenetics at diagnosis, and this may be a reason why high‐risk cytogenetic abnormalities did not emerge as significant prognostic factors in the multivariate analysis. Advanced imaging techniques, including whole‐body low‐dose computed tomography, were introduced after 2000 in clinical routine. All patients had at least one of the conventional CRAB criteria, as they were diagnosed before 2010, and the introduction of the SLiM criteria occurred after 2014 [56].

The introduction of novel agents and next‐generation drugs both in the upfront and in the relapsed and/or refractory setting during the last decade has further improved the disease course [3, 39]. Therefore, a much greater proportion of patients who are currently diagnosed with symptomatic MM and receive quadruplets (an anti‐CD38 monoclonal antibody—daratumumab or isatuximab, a proteasome inhibitor—bortezomib or carfilzomib, an immunomodulatory drug—lenalidomide, and dexamethasone) are anticipated to reach at least 15 years of PFS in the near future [57].

In conclusion, among patients diagnosed and treated in the era before the availability of the contemporary new agents in the frontline setting, about 15% were long‐term survivors for > 15 years. Furthermore, about 8% remained in long‐term remission for more than 15 years even without continuous treatment. These patients were young at the time of initial MM diagnosis, had mostly low‐risk disease, and received ASCT with fixed‐duration maintenance as part of their primary therapy.

## Author Contributions

M.A.D. and E.K. contributed to the study conception and design. Material preparation, data collection, and analysis were performed by I.N.‐S., D.F., F.T., E.S., V.S., P.M., E.P., S.G., N.K., V.K., M.R., A.A., M.M., E.E.P., M.G., and E.T. The first draft of the manuscript was written by M.A.D. and I.N.‐S., and all authors reviewed and provided feedback. All authors read and approved the final version of the manuscript.

## Ethics Statement

All procedures performed in studies involving human participants were in accordance with the ethical standards of the institutional and/or national research committee and with the 1964 Helsinki declaration and its later amendments or comparable ethical standards.

## Conflicts of Interest

The authors declare no conflicts of interest.

## Data Availability

The data that support the findings of this study are available on request from the corresponding author.
